# A Song of Ice and Fire: Cold and Hot Properties of Traditional Chinese Medicines

**DOI:** 10.3389/fphar.2020.598744

**Published:** 2021-01-19

**Authors:** Juan Liu, Wuwen Feng, Cheng Peng

**Affiliations:** ^1^ School of Pharmacy, Chengdu University of Traditional Chinese Medicine, Chengdu, China; ^2^ State Key Laboratory of Characteristic Chinese Medicine Resources in Southwestern China, Chengdu University of Traditional Chinese Medicine, Chengdu, China

**Keywords:** clinical application, antipyretic effect, thermogenesis, cold and hot properties, traditional Chinese medicines

## Abstract

The theory of cold and hot properties is the basic theory of traditional Chinese medicines (TCMs) and has been successfully applied to combat human diseases for thousands of years. Although the theory of cold and hot is very important to guide the clinical application of TCMs, this ancient theory remains an enigma for a long time. In recent years, more and more researchers have tried to uncover this ancient theory with the help of modern techniques, and the cold and hot properties of a myriad of TCMs have been studied. However, there is no review of cold and hot properties. In this review, we first briefly introduced the basic theories about cold and hot properties, including how to distinguish between the cold and hot properties of TCMs and the classification and treatment of cold and hot syndromes. Then, focusing on the application of cold and hot properties, we take several important TCMs with cold or hot property as examples to summarize their traditional usage, phytochemistry, and pharmacology. In addition, the mechanisms of thermogenesis and antipyretic effect of these important TCMs, which are related to the cold and hot properties, were summarized. At the end of this review, the perspectives on research strategies and research directions of hot and cold properties were also offered.

## Introduction

Traditional Chinese medicines (TCMs), an important category of complementary and alternative medicines, have been widely used in China and the surrounding areas for thousands of years (Zhang Q. et al., 2016). Because of the rich experience in combating various diseases and the increasing acceptance of complementary and alternative medicines, there is a growing trend to use TCMs as a therapeutic option to treat diseases in some Western countries including the United States, the United Kingdom, and Australia (Cheung, 2011; Lai et al., 2017; Porter et al., 2017; Chao et al., 2020). One of the characteristics of TCM is that the traditional Chinese culture and philosophy such as *Yin* and *Yang* are rooted in TCM (Huang et al., 2018), and thus TCM contains unique theories to guide the clinical application of TCMs. Those theories include cold and hot properties, five flavors, compatibility (combinational use of TCMs), and so on (Zhang J. H. et al., 2015; Wang Y. et al., 2016). For a TCM practitioner, he or she must obey those theories because violation of those theories would lead to the increase of toxicity or reduction of efficacy (Wang S. P. et al., 2012). For example, the combination of Gancao (the dried root and rhizome of *Glycyrrhiza uralensis* Fisch. ex DC.) and Gansui (the dried root of *Euphorbia kansui* S.L.Liou ex S.B.Ho) will cause side effects or even toxicity (Shen et al., 2016).

Among all the TCMs theories, the theory of cold and hot properties plays a fundamental role in the clinical practice of TCMs. This theory was first recorded in *Shennong Bencao Jing* (200–300 AD, Han Dynasty) and has been used by traditional Chinese practitioners to treat diseases for thousands of years. According to the rules of cold and hot properties recorded in *Shennong Bencao Jing*, patients with cold syndrome should be treated by TCMs with hot property and patients with hot syndrome should be treated by TCMs with cold property. The typical TCMs with cold property, like Zhimu (the dried rhizome of *Anemarrhena asphodeloides* Bunge) and Huanglian (the dried rhizome of *Coptis chinensis* Franch.), are used to relieve hot syndrome (Chen H. et al., 2015; Ota et al., 2019; Yu et al., 2020). And TCM practitioners applied TCMs with hot property, such as Fuzi (the processed lateral root of *Aconitum carmichaelii* Debeaux), to remedy cold syndrome (Rong et al., 2016). Even though thousands of years of clinical experience and modern pharmacological studies have proved the efficacy of those TCMs with strong cold or hot property, according to the book *Yizong Bidu* (written by Li Zhongzi in the Ming dynasty), the violation of the application rules of cold and hot properties is not permitted because such a violation may lead to the failure of therapy and even aggravate the symptoms. For example, a coughing patient with hot syndrome has taken hot TCMs (the dried stem of *Ephedra sinica* Stapf, the twigs of *Cinnamomum cassia* (L.) J.Presl, the dried *Zingiber officinale* Roscoe, etc.) and finally exhibited dry mouth, nose and throat, persistent cough, excessive phlegm, and other hot syndromes, which is a typical case of hot TCMs being misused for hot syndrome resulting in hot syndrome aggravation (Zhang and Feng, 1987). Therefore, only when the medical practitioner has mastered the cold and hot properties, can the safety and effectiveness of TCMs be ensured.

**FIGURE 1:**
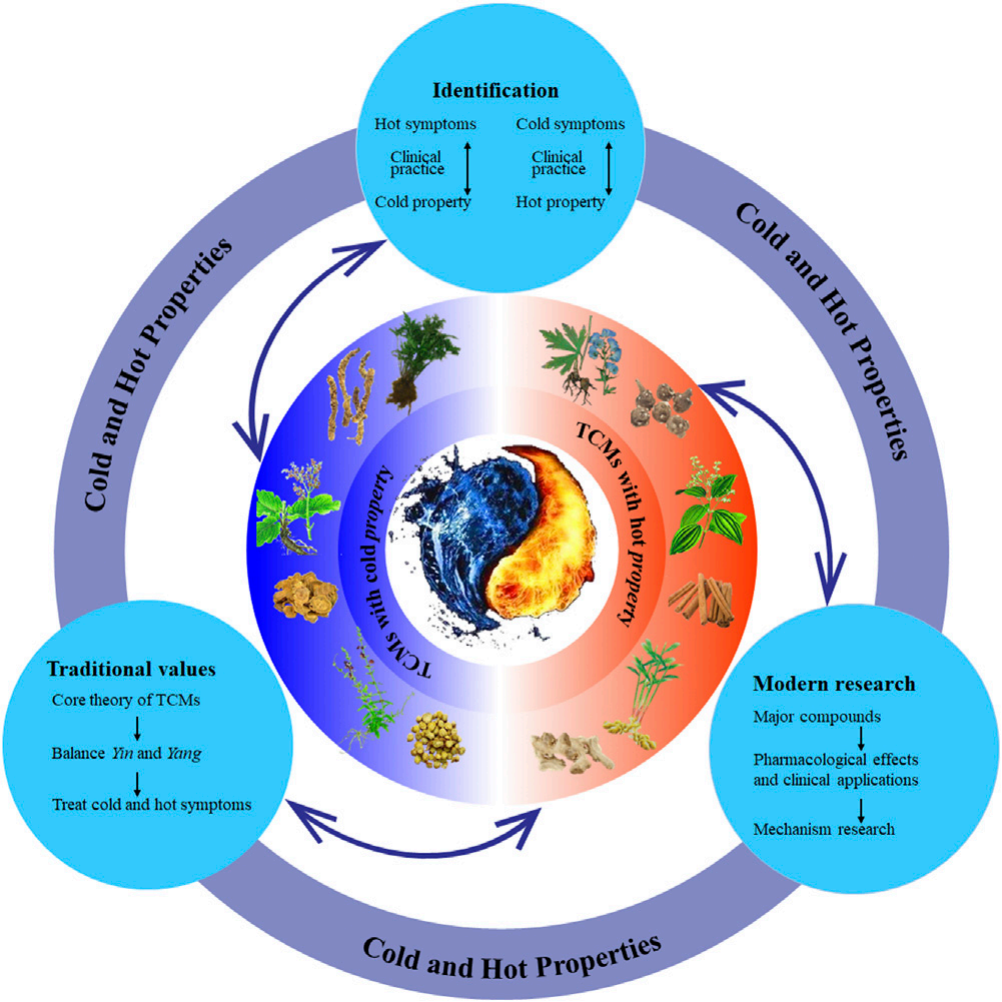
The overall picture to understand the cold and hot properties of TCMs. TCMs, traditional Chinese medicines.

As the core theory of TCM, cold and hot properties have been studied by more and more researchers due to its important role in guiding clinical diagnosis and treatment. With the development of new techniques, more and more efforts have been made in recent years to identify the cold and hot properties of TCMs, classify cold syndrome and hot syndrome, and study the effects of cold and hot TCMs on hot syndrome and cold syndrome. In addition, an increasing number of studies have focused on phytochemistry and pharmacology to investigate corresponding compounds associated with cold and hot TCMs and the mechanism of cold and hot TCMs. Although achievements have been made in the research of cold and hot properties, there has been no review of cold and hot properties. In this review, we summarized and discussed the studies of the cold and hot properties of TCM (Figure 1). We hope this review of hot and cold properties will help researchers to understand the art and science of cold and hot properties of TCM and finally to promote the rational use of TCM.

## The Identification of Cold and Hot Properties of Traditional Chinese Medicines

The identification of cold and hot properties mainly depends on the healthy human’s direct feeling on a given TCM or the effects of TCMs on patients with cold or hot syndrome. For example, masticating mint leaves will produce a cold feeling, while chewing a piece of ginger will cause a hot feeling (Yu et al., 2020). Therefore, the mint leaf is classified as a cold TMC and ginger is classified as a hot TCM. When a person is ill, he or she may feel cold or hot and exhibit cold or hot syndrome. In general, the TCMs that can reduce or eliminate hot syndrome in a patient are cold in property. For example, Zhimu (the dried rhizome of *Anemarrhena asphodeloides* Bunge) and Zhizi (the dried ripe fruit of *Gardenia jasminoides* J.Ellis) are able to treat hot syndromes such as high fever, sweating, thirst, and powerful femtosecond pulse, so the properties of the two TCMs are cold (Chen, et al., 2005). On the contrary, the TCMs that can alleviate or eliminate cold syndrome are ordinarily hot in property. For instance, Fuzi (the processed lateral root of *Aconitum carmichaelii* Debeaux) and Rougui (the dried stem bark of *Cinnamomum cassia* (L.) J.Presl) are capable of treating chills, blood stasis, pain, stomachache, head, and pulse floating; thus, these two TCMs are hot in property (Zhou B. C. et al., 2019; Wang J. et al., 2020).

Although the theory of hot and cold properties is very important in clinical practice, this theory remains to be questioned because this theory mainly depends on human’s sensory response and lacks scientific evidence. In recent decades, researchers have made a lot of efforts in animal models for studying the cold and hot properties of TCMs. These models for studying the cold and hot properties of TCMs can be divided into three types: animal models made with TCMs, biochemical or chemical drug-induced model, and cold exposure-induced model. For example, the cold syndrome induced by Huangbai (the dried stem bark of *Phellodendron chinense* C.K.Schneid.), Zhimu (the dried rhizome of *Anemarrhena asphodeloides* Bunge), etc. and the hot syndrome induced by Fuzi (the processed lateral root of *Aconitum carmichaelii* Debeaux), Rougui (the dried stem bark of *Cinnamomum cassia* (L.) J.Presl), etc. were used to study the properties of TCMs (Han X. Y. et al., 2018). Li et al. used collagen-induced arthritis rat model to explore the effect of TCM formulas with cold and hot properties on the ultrastructures of synoviocytes (Li et al., 2002). In addition, cold-stress-induced hypothermia mice were used to study the effect of Fuzi (the processed lateral root of *Aconitum carmichaelii* Debeaux, a hot TCM) on hypothermia (Makino et al., 2009). With the development of new techniques, more and more efforts have been made in recent years to characterize the cold and hot properties of TCMs. Those methods include but not limited to microcalorimetry (Chen Z. et al., 2015), monitoring of animal thermotropism behavior (Zhao et al., 2011), cell temperature measurement (Yu et al., 2020), chemical space analysis (Fu et al., 2017), statistical pattern recognition (Wang X. Y. et al., 2012), bioinformatics analysis and chemical structure analysis (Liang et al., 2013), multisolvent similarity measurement (Wei et al., 2019), gene expression profile (Li A. R. et al., 2020), metabolomics, and network pharmacology (Li et al., 2014; Xia et al., 2020). For example, based on the fact that the temperature of a living organism is directly related to energy production, Xiao et al. established a method for assessment of the hot properties of Fuzi (the processed lateral root of *Aconitum carmichaelii* Debeaux) according to the biothermodynamics parameters such as bacterial growth rate and energy release (Chen Z. et al., 2015). At the animal level, Zhao et al. studied the cold and hot properties by observing the influence of TCMs on animal behaviors and functions, and it was found that cold TCMs enhanced thermotropism in animals and reduces energy metabolism, while hot TCMs had the opposite effects (Zhao et al., 2011). In short, animal model and modern technology have played important roles in the identification of cold and hot properties.

The cold and hot properties of TCMs mainly consist of four subtypes, including warm, hot, cold, and cool (Wang Y. et al., 2016). For a warm TCM, it is classified as a hot TCM, but its hot property is not strong. Similarly, for a cool TCM, it is classified as a cold TCM, but its cold property is not strong. It should be noticed that although the cold and hot properties of most TCMs can be easily identified, the cold and hot properties of some TCMs are not clear because they do not exhibit obvious cold and hot properties. And those TCMs are usually accepted as neutral TCMs. For example, TCMs such as Fuling (a fungus, the dried sclerotium of *Poria cocos* (Schw.) Wolf) and Dangshen (the dried root of *Codonopsis pilosula* (Franch.) Nannf.) are not classified as cold TCMs or hot TCMs, but neutral TCMs (Ung et al., 2007). Because neutral TCMs have no obvious effect on the clinical manifestations that are related to cold and hot syndromes, there are some challenges in the study of neutral TCMs, leading to few studies on neutral property. In this context, it is currently difficult to summarize the studies of neutral TCMs, especially the mechanism of neutral TCMs. Therefore, this review mainly focuses on the hot and cold properties of TCMs.

## The Classification and Treatment of Cold and Hot Syndromes

As a basic unit and the key concept in TCM theory, syndrome (ZHENG in Chinese) is a holistic summary of the patient’s status and has been used to diagnose and treat disease for thousands of years (Li et al., 2007; Kanawong et al., 2012; Su et al., 2012). TCM practitioners distinguish between the conditions of individual patients according to the TCM syndrome. The cold syndrome and hot syndrome are the two most common and representative syndromes that represent two opposite but interrelated conditions of the human body (Zhou N. et al., 2019). Tongue appearance is a valuable diagnostic tool for determining syndrome in patients. According to the color and texture of the patient’s tongue coating, the cold syndrome and hot syndrome are identified by a white-greasy and yellow-dense tongue coating, respectively (Jiang et al., 2012; Cui et al., 2019). In addition to tongue coating symptoms, cold syndrome and hot syndrome also show other symptoms. The cold syndrome also shows symptoms such as hypothermia, cold limbs, lost appetite, diarrhea, nausea, and vomiting (Mei, 2011). On the contrary, the hot syndrome generally has symptoms including high fever, unconsciousness, delirium, dysphoria, thirst, constipation, oral ulcer, sore in mouth, and dry eye (Mei, 2011; Zhang, 2011). According to this classification, TCMs with hot property are used to treat cold syndrome, whereas cold TCMs are used to treat hot syndrome (Zhou N. et al., 2019). For example, the representative cold TCMs, such as Huanglian (the dried rhizome of *Coptis chinensis* Franch.), Huangqin (the dried root of *Scutellaria baicalensis* Georgi), and Dahuang (the dried root and rhizome of *Rheum palmatum* L.), are extensively used for the treatment of hot syndrome (Xiao et al., 2019), whereas the typical hot TCMs, such as Fuzi (the processed lateral root of *Aconitum carmichaelii* Debeaux), Ganjiang (the dried *Zingiber officinale* Roscoe), and Rougui (the dried stem bark of *Cinnamomum cassia* (L.) J.Presl), are widely used to treat cold syndrome (Panthi et al., 2017). These cold TCMs and hot TCMs can be combined to form cold and hot formulas (Fu-Fang in Chinese) according to compatibility principle. These formulas in TCM also play a prominent role in the treatment of cold and hot syndromes. For example, one such formula for hot syndrome is the *Gegen Qinlian Decoction*, an ancient and effective treatment for dampness hot syndrome including diarrhea and dysentery, which originated from *Shanghan Lun* that is a canonical book of TCM compiled by Zhang Zhongjing (Li et al., 2014). And the classical TCM formula, *Liuwei Dihuang Pills*, with the function of nourishing kidney yin, is the major formula for Xiao Ke disease of yin deficiency and internal heat (Zheng et al., 2019). Moreover, *Lily Bulb and Rehmannia Decoction* has long been used as tonics for nourishing heart and lung, clearing heat and cooling blood in the treatment of mental instability, absent mindedness, dysphoria, and depression (Chi et al., 2019; Zhang et al., 2019; Zhang et al., 2020). The above three examples are typical cases of TCM formulas for the treatment of hot syndrome. For cold syndrome, *Sini Decoction*, a classical TCM formula recorded in *Shanghan Lun*, has a significant therapeutic effect on cold symptoms displaying coldness on the extremities, vomiting, and diarrhea and is considered to be a therapy that has the essential effect of recuperating the patients from collapse (Cheng et al., 2016). Therefore, cold TCMs and hot TCMs, including single herb and formula, are indispensable for the treatment of cold and hot syndromes.

In addition to single cold syndrome or hot syndrome, patients often suffer a cold-hot complicated syndrome (Kemler et al., 2001). Patients with this syndrome can manifest cooling of limbs (representation of cold syndrome) and red tongue (representation of hot syndrome). Because the etiology and pathogenesis of cold-hot complicated syndrome are complicated, it is difficult to treat this syndrome by using cold TCMs or hot TCMs alone. For this complicated syndrome, formulas composed of cold and hot TCMs can be used (Chen et al., 2018). *Shanghan Lun*, a canonical book of TCM written by Zhang Zhongjing, contains a large number of examples of combinational use of cold and hot medicines, such as *Banxia Xiexin Decoction*, *Xiao Chaihu Decoction* (Shao and Li, 2019), and *Wumei Pill* (Yan et al., 2012). According to TCM theory, when formula composed of cold and hot TCMs is adopted, the hot syndrome can be treated by cold TCMs, and the cold syndrome can be treated by hot TCMs, eventually eliminating cold-hot complicated syndrome. Therefore, for cold-hot complicated syndrome, formulas composed of cold and hot TCMs are essential.

**FIGURE 2:**
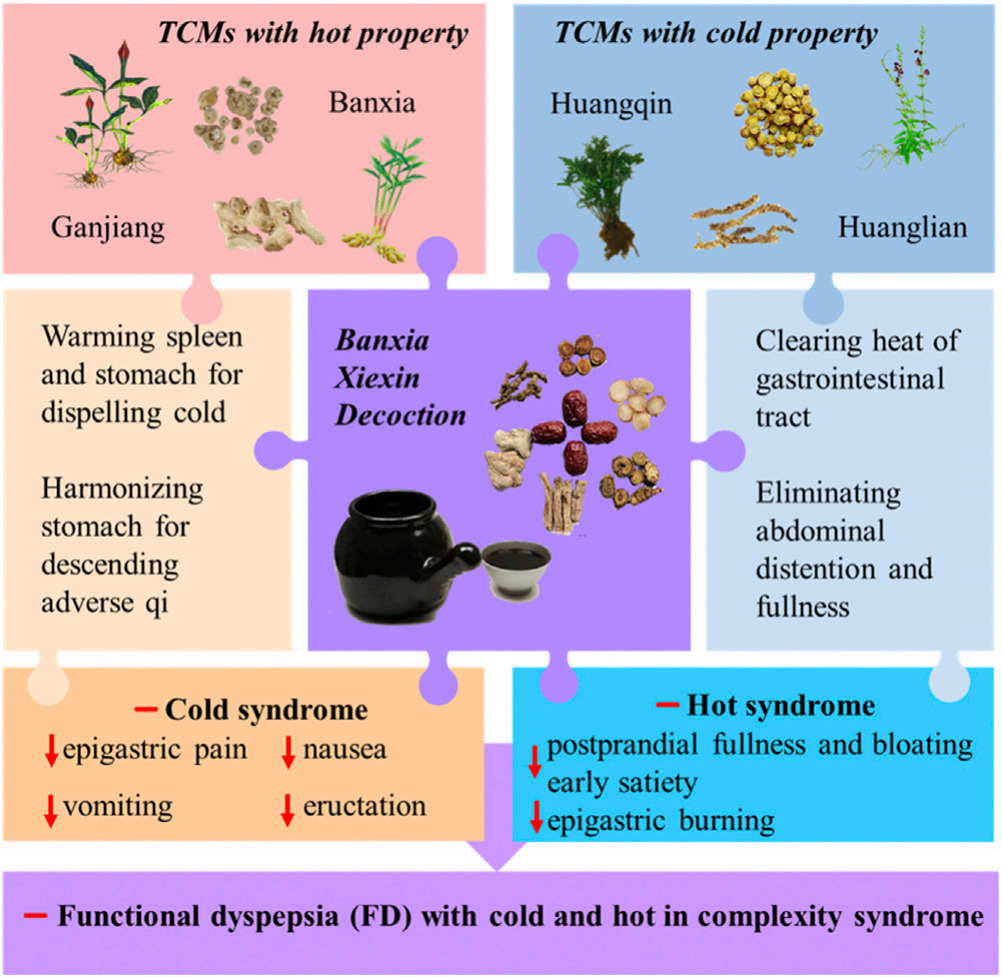
*Banxia Xiexin Decoction* for the treatment of functional dyspepsia with cold and hot complexity syndrome. TCMs, traditional Chinese medicines; ↓, decrease; −, inhibition.

One such formula for cold-hot complicated syndrome is the *Banxia Xiexin Decoction*. It is an ancient and effective treatment for diarrhea (hot syndrome) and vomiting (cold syndrome) (Min, 2009; Zhao and Song, 2011). In the formula, Huanglian (the dried rhizome of *Coptis chinensis* Franch.) and Huangqin (the dried root of *Scutellaria baicalensis* Georgi) with cold property can clear heat and stop diarrhea; meanwhile, Banxia (the dried tuber of *Pinellia ternata* (Thunb.) Makino) and Ganjiang (the dried *Zingiber officinale* Roscoe) with hot property have the functions of eliminating cold and stopping vomiting. A randomized controlled trial involving patients from five centers evaluated the efficacy of modified *Banxia Xiexin Decoction* in the treatment of functional dyspepsia (FD) with cold-hot complicated syndrome. After 4 weeks of treatment, the results showed that, compared with the placebo group, the modified *Banxia Xiexin Decoction* significantly improved epigastric pain, postprandial fullness and bloating, early satiety, and a burning sensation in the stomach, indicating that it had a significant improvement effect on the FD with cold-hot complicated syndrome (Zhao et al., 2013a) (Figure 2). Another typical example involving cold-hot complicated syndrome is rheumatoid arthritis (RA). Many patients with RA show the hot symptoms including dry mouth, thirst, fever, and restlessness, as well as the cold symptoms including stiff joints, stabbing pain, sharp pain, and cold aggravated pain (Regestein, 2012). *Guizhi-Shaoyao-Zhimu Decoction* (GSZD), a formula composed of 9 TCMs such as Guizhi (the dried bark of *Cinnamomum cassia* (L.) J.Presl, hot in property) and Zhimu (the dried rhizome of *Anemarrhena asphodeloides* Bunge, cold in property) has been extensively used in the treatment of RA. Research has shown that GSZD can significantly improve cold syndromes (such as pain) and hot syndromes (such as redness and swelling) in RA (Guo et al., 2016). The above studies have further confirmed that the formulas composed of cold and hot TCMs have a clear therapeutic effect on cold-hot complicated syndrome. However, the mechanisms of these formulas in the treatment of cold-hot complicated syndrome is still unclear.

In recent years, there has been increasing concern about the application of bioinformatics and systems biology approaches to understand the development of diseases and the mechanism of the TCMs (Li and Zhang, 2013; Zheng et al., 2018; Tan et al., 2019; Wang X. et al., 2020). Based on the new trend in interdisciplinary fields, Li et al. established TCM systems bioinformatics (TCMSB) and proposed for the first time a map of “Phenotype network-Biological network-Herb network,” which contributes to identifying network biomarkers for TCM syndrome and herb formula (Li et al., 2007; Li, 2009). In this context, the relationship between cold-hot complicated syndrome and formula could be transferred into a network context. For example, Li et al. (2007) investigated cold syndrome and hot syndrome in the context of the neuro-endocrine-immune (NEI) system and identified the network biomarkers for both syndromes. They found that hormone-related genes were predominant in the cold syndrome network, immune-related genes were predominant in the hot syndrome network, and neurotransmitter-related genes were distributed in both cold syndrome network and hot syndrome network. Therefore, TCM network pharmacology can be helpful to decipher the molecular mechanisms of TCM formulas for treating cold-hot complicated syndrome.

## The Important Hot and Cold Traditional Chinese Medicines and Their Scientific Application

As the cold and hot properties of TCMs are regarded as the basic elements of the clinical practice of TCMs, their scientific applications have been in the spotlight from the researchers for a long time. Here, we took the major TCMs with hot property or cold property, namely, Fuzi (the processed lateral root of *Aconitum carmichaelii* Debeaux), Ganjiang (the dried *Zingiber officinale* Roscoe), Rougui (the dried stem bark of *Cinnamomum cassia* (L.) J.Presl), Huanglian (the dried rhizome of *Coptis chinensis* Franch.), Dahuang (the dried root and rhizome of *Rheum palmatum* L*.*), and Huangqin (the dried root of *Scutellaria baicalensis* Georgi) as examples to demonstrate the research progress on cold and hot properties of TCMs. These TCMs are taken as representatives for serval reasons. First, the cold property or hot property of these important TCMs is very clear according to the canonical books such as Shennong Bencao Jing. Secondly, these TCMs are frequently used in clinic and have prominent therapeutic effects on cold syndrome or heat syndrome. Thirdly, in the modern research of cold and hot properties of TCMs, these TCMs are often taken as typical research objects, and a large number of research achievements on the cold and hot properties of these TCMs have been accumulated.

### Important Hot Traditional Chinese Medicines for Cold Syndrome

#### Fuzi

Fuzi, the processed lateral root of *Aconitum carmichaelii* Debeaux (Ranunculaceae), is an herb extensively used in TCM to treat the diseases with cold symptoms because of its hot property, which was originally recorded in *Shennong Bencao Jing* (Zheng et al., 2014; Li C. et al., 2020)*.* In *Bencao Jingdu*, it was viewed as the key TCM to ameliorate *Yang* deficiency and rescue the patient from prostration (Zhou et al., 2015). Interestingly, *Huoshen Pai*, a school of TCM, has become famous for the use of large doses of Fuzi to enhance *Yang* (Hong, 2012), which highlighted the importance of Fuzi in the treatment of cold syndrome.

Due to Fuzi’s efficacy of reinforcing *Yang*, dispelling cold, and relieving pain, it was usually used to treat *Yang* depletion syndrome (a moribund syndrome with almost no appearance of *Yang*), *Yang* deficiency syndrome, and cold pain. *Sini Decoction* consisting of Fuzi, Ganjiang (the dried *Zingiber officinale* Roscoe), and Gancao (the dried root and rhizome of *Glycyrrhiza uralensis* Fisch. ex DC.), recorded in *Shanghan Lun*, is the most typical formula to illustrate the traditional usage of Fuzi. It can revive *Yang* and is suitable for cold symptoms such as prostration, cold sweat, and cold limbs, as well as weak and impalpable pulse. *Shenfu Decoction* composed of Fuzi and Ginseng (the dried root and rhizome of *Panax ginseng* C.A.Mey.) is able to invigorate qi and restore *Yang* and is generally used to improve energy loss, cold extremities, sweating, plus feeble, and impalpable pulse, which originated from *Shengji Zonglu*. *Sini Decoction* and *Shenfu Decoction* are two representative formulas for the treatment of *Yang* depletion syndrome. Their application fully illustrates that Fuzi is the essential TCM to restore *Yang* and rescue the patient from collapse. In addition, Fuzi can also be applied to treat *Yang* deficiency syndrome (deficiency of spleen *Yang*, kidney *Yang*, and heart *Yang*) and cold-coagulation pain. For example, *Fuzi Lizhong Pill* plays an important role in warming the middle-jiao and invigorating the spleen; thereby, it is favorably applied for patients with the deficient cold in spleen and stomach, cold pain in the abdomen, vomiting, diarrhea, and cold in limbs, which was recorded in *Taiping Huimin Heji Jufang*. For the deficiency of kidney *Yang*, *Zhenwu Decoction* in *Shanghan Lun*, composed of Fuzi, Fuling (a fungus, the dried sclerotium of *Poria cocos* (Schw.) Wolf; the dried sclerotium of *Poria cocos* (Schw.) Wolf), Baishao (the dried root of *Paeonia lactiflora* Pall.), Baizhu (the dried rhizome of *Atractylodes macrocephala* Koidz.), and so on, is an appropriate choice as it can warm and enhance kidney *Yang* to promote diuresis and improve swelling of the body. For the aspect of cold-coagulation pain, *Gancao Fuzi Decoction*, composed of Fuzi, Gancao (the dried root and rhizome of *Glycyrrhiza uralensis* Fisch. ex DC.), Baizhu (the dried rhizome of *Atractylodes macrocephala* Koidz.), and Guizhi (the dried bark of *Cinnamomum cassia* (L.) J.Presl), has cure function to wind-cold-dampness arthralgia syndrome. Taken together, these traditional usages of Fuzi further demonstrated its potent role in the treatment of cold syndrome, including *Yang* depletion syndrome (a moribund syndrome with almost no appearance of *Yang*), *Yang* deficiency syndrome, and cold pain.

Because Fuzi has a prominent effect on the treatment of cold syndrome, many modern studies have been carried out around it to explore the mystery of the traditional function of Fuzi. Phytochemical research has indicated that alkaloids such as aconitine, mesaconitine, hypaconitine, benzoylmesaconine, benzoylaconitine, and benzoylhypacoitine are the main bioactive constituents and the quality markers of Fuzi (Chen et al., 2008; Tang et al., 2012; Yu et al., 2015). In addition to alkaloids, a fraction of other ingredients has also been isolated and identified, including flavonoids, glucides, saponins, glycosides, fatty acids, ceramides, and uracil (Konno et al., 1985; Sang et al., 2005; Lyu et al., 2008; He et al., 2018; Sun et al., 2019; Zhao et al., 2020). The pharmacologic activities of Fuzi included, but were not limited to, thermogenesis (Deng et al., 2019); the effects on the cardiovascular system (Zhao et al., 2012), energy metabolism (Zheng et al., 2014), kidney protection (Tan et al., 2014), and immune system, anti-inflammation and analgesic action (Yang et al., 2020), antitumor (Zhang et al., 2017), hypoglycemic, and hypotensive effects, etc. (Huang et al., 2010). Based on the hot property and pharmacological effects of Fuzi, nowadays, it is used in clinical prescriptions for the treatment of shock, hypotension, coronary heart disease, chronic heart failure, diarrhea, syncope, neuralgia, joint pain, and rheumatoid arthritis (Zhou et al., 2015; Zuo et al., 2015).

Since the thermogenesis of Fuzi is closely related to its hot property, the research of the mechanism of thermogenesis of Fuzi will help explain the hot property of Fuzi. From the perspective of body temperature, the water extract of Fuzi was founded to significantly restore the decrease in core body temperature in hypothermic mice induced by cold stress. Further research found that Fuzi can enhance the production of heat by increasing the expression of uncoupling protein 1 (UCP1), a protein that can mediate heat generation in brown fat tissue (BAT) (Makino et al., 2009; Harms and Seale, 2013). Specifically, peroxisome proliferator-activated receptors (PPARs) mediate the thermogenesis of Fuzi. The expressions of PPAR gamma (PPAR-*γ*) and PPAR coactivator 1-alpha (PGC-1*α*) were upregulated by Fuzi treatment, and the activation of PPAR-*γ* further stimulated the UCP1 expression (Makino et al., 2009). In addition, the hot property of Fuzi can be partially explained by its effect on energy metabolism. At the cellular level, Fuzi-containing serum could significantly increase Na^+^-K^+^-ATPase and Ca^2+^-Mg^2+^-ATPase activities in hepatocytes, as well as intracellular ATP content, then promote cell energy metabolism, and increase intracellular energy reserves (Kan et al., 2018). Wang et al. found that the heat generation of rats could be increased by the water extract of Fuzi, and this process is associated with modulation of sugar metabolism, fatty acid metabolism, pentose phosphate pathway, and amino acid metabolism pathway (Wang et al., 2015). All these studies showed that the thermogenic mechanism of Fuzi involves the activation of BAT and the promotion of energy metabolism.

#### Ganjiang

Ganjiang (the dried *Zingiber officinale* Roscoe, or dried ginger in Chinese), another typical hot TCM, has been used as a food and herbal medicine in China, India, and other southeast Asian countries for thousands of years (Zhou et al., 2017). Ganjiang was first recorded in *Mingyi Bielu* in the Han Dynasty and it was described as a TCM with strong hot property (Chang et al., 1995; Fang et al., 2017; Zhou et al., 2017). Interestingly, Ganjiang can influence the hot property of Fuzi. A study based on animal thermotropism behavior found that, compared with the Fuzi group, the combination of Ganjiang and Fuzi significantly reduced the time and distance of mice moving on the warm plate, which indicated that Ganjiang enhanced the hot property of Fuzi (Sun et al., 2012).

In view of the efficacy of Ganjiang, it is widely used in prescriptions to treat patients with cold syndrome. *Shanghan Lun* and *Jingui Yaolüe* were respected as the ancestors of the prescriptions. Among them, numerous representative prescriptions containing Ganjiang are still widely used today for the treatment of cold syndrome. For example, the *Sini Decoction* in *Shanghan Lun*, a prescription composed of Ganjiang, Fuzi, and Gancao (the dried root of *Glycyrrhiza uralensis* Fisch. ex DC.), is one of the most classic formulas in a series of prescriptions containing Ganjiang. The combination of Ganjiang and Fuzi can not only increase the efficacy of Fuzi to restore *Yang* deficiency symptoms that manifested as prostration, deadly cold hand and foot, and weak pulse but also reduce the toxicity of Fuzi. For syndrome of deficient cold of spleen and stomach, *Lizhong Pill* in *Shanghan Lun*, a formulation composed of Ganjiang, Ginseng (the dried root and rhizome of *Panax ginseng* C.A.Mey.), and Baizhu (the dried rhizome of *Atractylodes macrocephala* Koidz.) has the function of invigorating spleen and qi. Thus, it can treat cold syndromes such as cold pain of epigastric abdomen, loss of appetite, reduced diet, vomiting, and diarrhea. Furthermore, *Banxia Ganjiang San*, a classical preparation recorded in *Jingui Yaolüe* and composed of Ganjiang and Banxia (the dried tuber of *Pinellia ternata* (Thunb.) Makino), has strong effects of dispelling cold and preventing vomiting. Because of the powerful effect of dispersing cold in the spleen and stomach, Ganjiang is regarded as a drug with broad-spectrum antiemetic effect (Oh, 2003). The cold-dispelling function of Ganjiang can also relieve pain. Combinational use of Ganjiang and TCMs with hot property can enhance effect of Ganjiang to dispel cold and relieve pain. *Erjiang Pill* in *Heji Ju Fang*, consisting of Ganjiang and Gaoliangjiang (the dried rhizome of *Alpinia officinarum* Hance), exerts potent function of dispersing cold to improve pain in the heart and spleen. In addition, Ganjiang has the function of warming lung and can be used to treat cough and asthma caused by cold, cold in the back, and phlegm retention. For example, *Xiaoqinglong Decoction* consisting of Ganjiang, Xixin (the dried root and rhizome of *Asarum heterotropoides* F.Schmidt), Wuweizi (the dried ripe fruit of *Schisandra chinensis* (Turcz.) Baill.), Mahuang (the dried stems of *Ephedra sinica* Stapf), etc. is very effective for patients with fluid retention, cough, and asthma, which is registered in *Shanghan Lun*.

Ginger contains about 58% oleoresin and 9% lipids or glycolipids. The spicy taste of ginger is mainly affected by the 25% pungent components of oleoresin that is mainly composed of gingerol. Ginger contains up to 3% volatile oils, accounting for 20%–25% of oleoresin (Chrubasik et al., 2005). More than 200 compounds have been identified from ginger, and its biologically active components include volatile oils, anthocyanins, tannins, and spicy compounds (gingerol, sesquiterpenes, and shogaols) (Semwal et al., 2015; Sharma et al., 2016). Moreover, volatile oil, gingerol, alkaloids, and diarylheptanoids were isolated from Ganjiang (Ajish et al., 2015; Li et al., 2016a; Shao et al., 2017). Previous studies have shown that the extracts and compounds of ginger have a variety of pharmacological activities, including thermogenesis (Miyamoto et al., 2015), hypoglycemic activity (Samad et al., 2017), antimicrobial (Ali et al., 2018), antiatherosclerosis (Wang S. et al., 2018), anti-inflammatory (Ho and Chang, 2018), analgesic (Hitomi et al., 2017), anticancer (Kapoor et al., 2016), antioxidant, antiaging functions (Han H. S. et al., 2018; Lee et al., 2019), the effect on the cardiovascular system and energy metabolism, sedative effect, and antiemetic activity (Vishwakarma et al., 2002; Yu et al., 2012; Wen et al., 2019). Correspondingly, Ganjiang is used clinically to treat patients with dizziness, vomiting caused by cold or during pregnancy cough, asthma, etc. (Wang W. X., 2016).

The hot property of Ganjiang can be partially explained by its thermogenic function. For example, Jiang Gui Fang (JG), composed of dried ginger and other TCMs, can increase core temperature, elevate the expressions of UCP1 and PGC-1*α* in BAT, and enhance the expressions of the lipogenic factor PPAR-*γ* and lipolytic protein hormone-sensitive triglyceride lipase (HSL) in white adipose tissue (WAT). *In vitro*, silent mating type information regulation 2 homolog 1 (SIRT1) was also enhanced by JG. Taken together, these results indicated that JG can activate BAT and induce browning of WAT via the PPAR*γ*/SIRT1-PGC1*α* pathway to increase thermogenesis (Zu et al., 2019). In addition, 6-capsaicin, the main active ingredient of Ganjiang, can promote the browning of adipocyte, which is proven by the increase of some brown/beige fat-specific genes such as PGC-1*α*, Cidea, PR domain containing 16 (Prdm16), gene encoding Cbp/p300-interacting transactivator 1 (Cited1), SIRT1, gene encoding transmembrane protein 26 (Tmem26), and Ucp1, as well as the increase of protein including UCP1, PGC-1*α*, and PRDM16. Some mitochondrial biogenesis markers such as SIRT1 and *p*-AMPK/AMPK are also increased by 6-capsaicin, which illustrates that 6-capsaicin improves mitochondrial respiration and energy metabolism (Wang et al., 2019).

#### Rougui

Rougui is the dried stem bark of *Cinnamomum cassia* (L.) J.Presl (Lauraceae) and can be used both as a condiment and as a drug. In America, it is used as a food supplement (Wang et al., 2013), while in Asia, it is often used as herbal medicine. In TCM, it is regarded as a Chinese medicine with strong hot property, and it was first recorded in *Shennong Bencao Jing* and listed as the top grade TCMs (Mamindla et al., 2017; Zhang et al., 2019).

According to the theory of TCMs, Rougui has the functions of warming *Yang*, dispersing cold to relieve pain, warming channels to promote blood circulation. To treat kidney *Yang* deficiency, Rougui is combined with other TCMs to enhance kidney *Yang.* Recorded in *Jingyue Quanshu*, *Yougui Pill*, a prescription containing Rougui and other TCMs such as Danggui (the dried root of *Angelica sinensis* (Oliv.) Diels) and Fuzi, can be used to treat patients with chilly limbs, cold pain in waist and knee, frequent urination, impotence, premature ejaculation, etc. For spleen and kidney *Yang* deficiency, *Guifu Lizhong Wan* (recorded in *Sanyin Fang*) consisting of Rougui, Fuzi, Ginseng (the dried root and rhizome of *Panax ginseng* C.A.Mey.), Baizhu (the dried rhizome of *Atractylodes macrocephala* Koidz.), etc. is used to treat symptoms such as cold limbs, decreased appetite, tiredness, and loose stools. For the symptoms of palpitations, shortness of breath, and chest tightness caused by heart *Yang* deficiency, Rougui is generally used in combination with TCMs such as Ginseng (the dried root and rhizome of *Panax ginseng* C.A.Mey.) and Huangqi (the dried root of *Astragalus mongholicus* Bunge) for warming *Yang* and supplementing qi. Rougui-containing prescriptions also play an important role in the treatment of cold-induced pain and blood stasis syndrome. For example, *Guifu Pills* consisting of Rougui, Ganjiang (the dried *Zingiber officinale* Roscoe), and so on have significant effects on treating chest pain and heartache caused by cold invasion, which was recorded in *Shoushi Baoyuan*. In addition, in *Beiji Qianjin Yaofang, Duhuo Jisheng Decoction* consisting of Rougui, Danggui (the dried root of *Angelica sinensis* (Oliv.) Diels), Sangjisheng (the dried twig of *Taxillus chinensis* (DC.) Danser), etc. has been shown to combat cold and dampness, so it is a commonly considered prescription for the treatment of rheumatism. For women with lumps in the abdomen due to qi stagnation and blood stasis, *Peng E Zhu Pill* in *Jiyin Gangmu*, mainly composed of Rougui, Ezhu (the dried rhizome of *Curcuma phaeocaulis* Valeton), and Taoren (the dried mature seed of *Prunus persica* (L.) Batsch), is a suitable choice because it can warm channels to promote blood circulation, thereby eliminating lumps.

Extensive research has been carried out on the phytochemical constituents of Rougui. More than 160 components have been isolated and identified from Rougui (Zhang et al., 2019). The chemical components in Rougui are divided into volatile and nonvolatile components, of which the volatile component (volatile oil) is the main active ingredient. Cinnamaldehyde, the main component of volatile oil of Rougui, is regarded as the quality marker of Rougui in Chinese pharmacopoeia (Zhang et al., 2019). In addition, Rougui contains some types of nonvolatile compounds such as polysaccharides (Liu X. et al., 2018), polyphenols (Anderson et al., 2004), and flavonoids (Pardede et al., 2017), and other ingredients such as coumarin (Kim, 2017). The wide range of pharmacological activities of Rougui and its components have been reported, including thermogenesis (Tamura et al., 2012), antioxidant, anti-inflammatory, antibacterial, antidiabetic, antiobesity (Song et al., 2017; Lv et al., 2017; Zhu et al., 2017; Wang Y. et al., 2018), antishock (Kwon et al., 2015), antithrombotic (Kontogiorgis et al., 2015), antigastric ulcer effect (Jung et al., 2011), and cardiovascular protection (Gao et al., 2015). Therefore, Rougui is mainly used in the treatment of cardiovascular diseases (Hwa et al., 2012), gastrointestinal diseases (Sebaia et al., 2019), diabetes (Shinjyo et al., 2020), kidney diseases (Zhao et al., 2013b), rheumatic diseases (Chen et al., 2019), gynecological diseases (Sun et al., 2016), etc.

Rougui, as a TCM with hot property, has an important thermogenic effect on human body, and the mechanisms are related to the thermogenic genes in adipose tissue. Kwan et al. showed that Rougui significantly enhanced the expression of UCP1 in 3T3-L1 adipocytes and subcutaneous adipocytes, as well as the expression of other brown adipocyte marker genes such as cell death-inducing DFFA-like effector A (*Cidea*), *Prdm16*, PPAR*γ*, PPAR*γ* coactivator-1 (*Pgc*), and the fatty acid oxidation marker gene carnitine palmitoyltransferase 1 (*Cpt1*). Rougui also significantly increased the mitochondrial protein biogenesis. Thus, it can induce the browning of subcutaneous adipocyte browning to promote thermogenesis (Shen et al., 2010; Kwan et al., 2017). Further research found that cinnamaldehyde (CA), the main active component of Rougui, activates thermogenesis through PKA/p38 MAPK signaling in subcutaneous adipocyte of mice (Jiang et al., 2017). Specifically, CA can increase the expression of heat production markers such as fibroblast growth factor 21 (*Fgf21*) and *Ucp1*, as well as activate PKA and subsequent phosphorylation of p38 MAPK, PKA-dependent phosphorylation of HSL, and lipid droplet-associated protein perilipin 1 (PLIN1). Furthermore, the PKA inhibitor H-89 can significantly block the phosphorylation of PKA substrate, p38 MAPK, HSL, and PLIN1. Correspondingly, the induction of *Fgf21* and *Ucp1* by CA was significantly inhibited by H-89 (Jiang et al., 2017).

### Important Cold Traditional Chinese Medicines for Hot Syndrome

#### Huanglian

Huanglian, dried rhizome of medicinal plants from the family Ranunculaceae such as *Coptis chinensis* Franch., *Coptis deltoidea* C.Y.Cheng and P.K.Hsiao, and *Coptis teeta* Wall., has been used in TCM for more than 2000 years (Yi et al., 2013; Qi et al., 2018). According to TCM theory, Huanglian is cold in property and bitter in taste and can enter the meridian of heart, stomach, large intestine, and liver and has the function of clearing away heat and dispelling dampness and detoxifying. It is often used in the treatment of damp-heat syndromes such as diarrhea and vomiting caused by gastrointestinal damp-heat, hot syndromes such as heart and stomach heat, and heat-toxicity syndromes such as carbuncle (Wang et al., 2014).

Specifically, for the hot syndrome of heart that manifested as fever, irritability, even delirium, Huanglian is often combined with the TCMs with the function of clearing heart fire or clearing heat and removing toxicity, such as *Huanglian Jiedu Decoction* in *Waitai Miyao*. In the treatment of dysphoria and palpitation caused by hyperactivity of heart fire, *Huanglian E-Jiao Decoction* in *Shanghan Lun*, mainly consisting of Huanglian, Ejiao (Colla Corii Asini), and Huangqin (the dried root of *Scutellaria baicalensis* Georgi), is often preferred. When heart fire is so intense that it causes hematemesis and bleeding, Huanglian is generally used in combination with the TCMs for cooling blood and arresting bleeding, such as *Xiexin Decoction* in *Jingui Yaolüe*. In addition, Huanglian also has a strong effect on clearing stomach fire. For example, *Qingwei San*, a prescription mainly composed of Huanglian, Shigao (CaSO_4_ • 2H_2_O), and other TCMs for clearing stomach heat, has been applied to treat the hot syndromes resulting in stomach fire, such as toothache, redness and swelling of gums, and bleeding of teeth. Of note is that, for the wasting syndrome (Xiao Ke Zheng) caused by stomach heat, including polydipsia, polyuria, polyphagia, emaciation, and fatigue, the powerful effect of Huanglian for clearing stomach fire has attracted much attention. Huanglian was viewed as a curative TCM for patients with wasting syndrome, which was recorded as early as the Wei and Jin Dynasties (1,500 years ago, 220–589 AD). In the subsequent dynasties, many records about Huanglian for the treatment of wasting syndrome were kept in the series of herbal classics. The *Xinxiu Bencao* in the Tang Dynasty (618–907 AD) pointed out that the Huanglian growing in western China is beneficial to the treatment of wasting syndrome. According to the analysis of *Taiping Shenghui Fang* (960–1279 A.D.), Huanglian is found to be one of the ten most commonly used TCMs in the treatment of wasting syndrome. Besides, in the *Puji Fang* completed in the Ming Dynasty around 1406 AD, 13 of the 64 formulas for treating wasting syndrome involved Huanglian. The *Bencao Gangmu* that was published in the same dynasty recorded the treatment of excessive urination, thirst, and emaciation with Huanglian. Therefore, the widespread use of Huanglian for the treatment of wasting syndrome has formed the foundation for its antidiabetic effect. In addition, because of the efficacy of Huanglian in removing damp-heat, clearing heat, and removing toxicity, it is often used to treat dysentery, sores, carbuncles, and furuncles, such as *Gegen Huangqin Huanglian Decoction* in *Shanghan Lun* and *Huanglian Jiuku Decoction* in *Waike Zhengzong*.

In 1862, berberine (BBR) was first reported as a chemical component isolated from Huanglian (Perrins, 1862). So far, more than 100 chemical components have been isolated and identified from it. Among them, alkaloids, including berberine, palmatine, coptisine, epiberberine, and magnoflorine, are the main active components. In addition to alkaloids, Huanglian also contains chemical components such as organic acids, coumarin, phenylpropanoids, and saccharides (Meng et al., 2018). The wide range of pharmacological effects of the extracts or compounds of Huanglian includes antipyretic, antibacterial, antiviral, antidiabetic, anticancer, anti-inflammatory, and cardiovascular protective effects (Liu et al., 2011; Tan et al., 2016; Hung et al., 2018; Ishikawa et al., 2018; Li et al., 2018; Liang et al., 2019; Kwon and Chan, 2020; Qin et al., 2020). In particular, the pharmacological effects of BBR have been extensively studied. Due to its outstanding antibacterial activity, BBR has been used as an over-the-counter (OTC) drug in the treatment of bacterial diarrhea in China for decades (Kong et al., 2004; Kumar et al., 2015; Jin et al., 2020). Since the early 2000s, BBR has been progressively seen as a potential medicine for hyperlipidemia and diabetes (Dong et al., 2012; Lan et al., 2015; Yao et al., 2015; Cicero and Baggioni, 2016). In addition to metabolic disorders, BBR has constructive effects against cardiovascular diseases, such as heart failure, arrhythmia, thrombosis, hypertension, atherosclerosis, and acute coronary syndrome (Lan et al., 2015; Yao et al., 2015; Li et al., 2016b; Wang K. et al., 2017).

Evidence for the antipyretic effect of Huanglian can partially account for this cold property of Huanglian. As a representative of heat-clearing drug, Huanglian appears in many classic prescriptions for heat-clearing. BBR, the main biologically active component of Huanglian (Zou et al., 2017), could inhibit the formation of arterial plaque and reduce the inflammatory response of aortic tissue in the atherosclerosis rats with damp-heat syndrome (Ke et al., 2020), which may be related to its antipyretic activity. As early as in 1971, Sabir et al. have studied the antipyretic effect of BBR by using a model of experimentally induced fever in rats (Sabir and Bhide, 1971). The antipyretic activity of BBR was also shown by another famous fever model, Brewer’s yeast-induced pyrexia mice, and the result was in line with previously reported by Sabir et al. (Küpeli et al., 2002). In order to further understand the potential mechanism of BBR in regulating body temperature, a recent study comprehensively investigated the effect of BBR on environmentally dependent thermogenesis in mice (Jiang et al., 2013). These results showed that BBR can antagonize increasing core body temperatures in hot environments, and further research showed that it limited the expression of HSP70 (heat shock protein 70) and TNF*α* (tumor necrosis factor, a proinflammatory cytokine) and in mice, suggesting that the antipyretic mechanism of BBR is related to heat shock protein and inflammatory cytokines (Jiang et al., 2013). In addition, Kong et al. demonstrated that Huanglian exerted antipyretic effect on yeast-induced pyrexia rats by regulating the expression of transient receptor potential vanilloid 1 (TRPV1, the activation of this protein will cause a burning sensation) and transient receptor potential melastatin 8 (TRPM8, a protein with the function of sensing cold). Specifically, Huanglian can inhibit febrile mediators such as prostaglandin E2 (PGE2) and cyclic adenosine 3′,5′-monophosphate (cAMP), increase antipyretics such as arginine vasopressin (AVP) to activate TRPM8, and inhibit TRPV1 in hypothalamic paraventricular nucleus and supraoptic nucleus, thus exerting antipyretic effect (Kong et al., 2014; Wan et al., 2014).

#### Dahuang

*Rheum palmatum* L., *Rheum tanguticum* (Maxim. ex Regel) Balf., and *Rheum officinale* Baill. (Polygonaceae) are endemic species to China (Zhou et al., 2014). Dahuang is their dried root and rhizome and is first recorded in *Shennong Bencao Jing*. As a representative TCM with cold property, Dahuang has obvious functions of relaxing bowels, clearing heat, detoxification, and cooling blood for hemostasis (Zhang R. Z. et al., 2015), and it has been widely used for cathartic, febrifugal, antidotal, and hemostatic purposes (Lee et al., 2003).

On the one hand, Dahuang can defecate stools and remove food that has accumulated in the gastrointestinal tract; on the other hand, it can clear away heat. Therefore, it is the representative TCM of Hanxia Fa (cold purgative method) (Xie, 2017). In *Shanghan Lun*, 113 of the 397 formulas mentioned the purgative method and 15 of the 113 formulas used Dahuang. And 20 prescriptions in *Jingui Yaolüe* selected Dahuang. In addition, more than 30 syndromes registered in *Wenyi Lun* can be treated by the purgative method, and Dahuang is used as the king TCM in these treatment programs. For instance, *Dachengqi Decoction* in *Shanghan Lun* selects Dahuang to treat constipation caused by heat accumulation. Dahuang in *Yinchenhao Decoction* helps to clear heat and treat jaundice induced by damp-heat, which is recorded in *Shanghan Lun*. In *Jingui Yaolüe*, the *Xiexin Decoction* consisting of Dahuang, Huanglian (the dried rhizome of *Coptis chinensis* Franch.), and Huangqin (the dried root of *Scutellaria baicalensis* Georgi) has an important effect on clearing away heat and cooling blood for stopping bleeding. Thereby, it has a good therapeutic effect on patients with hematemesis, bleeding, and hemoptysis. The *Dahuang Mudan Decoction* composed of Dahuang, Baishao (the dried root cortex of *Paeonia lactiflora* Pall.), etc. has the outstanding effect of clearing heat and detoxification, so it can treat abscess due to heat exuberance, which has been recorded in *Jingui Yaolüe*.

The main effective components of Dahuang are anthraquinones such as emodin, emodin methyl ether, aloe emodin, rhein, chrysophanol, etc. In addition, phenolic compounds were identified including catechins, glucose gallates, naphthalenes, sennosides, and stilbenes (Zargar et al., 2011; Pandith et al., 2014; Pandith et al., 2018). According to reports, the crude extracts and isolated compounds of Dahuang have a wide spectrum of pharmacological activities including antipyretic, antibacterial, antifungal, antiviral, anti-inflammatory, antioxidant, anticancer, hypoglycemic, immune-enhancing, liver-protective, kidney-protective, and central neuroprotective effect (Zargar et al., 2011; Kong et al., 2014; Esposito et al., 2016; Li X. et al., 2019). Dahuang’s medicinal history can be traced back to a long time ago, and it is known to be effective in treating nearly 57 different diseases (Rokaya et al., 2012). The extracts from Dahuang roots, skins, and leaves have been used as laxatives to help patients ameliorate constipation since ancient times (Wang et al., 2007). In addition, it can also be used in the prevention and treatment of inflammation, tinnitus, bleeding, pain, dysmenorrhea, tumors, diabetes, gastric ulcers, Parkinson’s disease, Alzheimer’s disease, depression, and severe acute respiratory syndrome (SARS) (Lakshmi, 2016; Li X. et al., 2019).

Notably, previous studies reported that emodin, a principle compound from Dahuang, could regulate cold- and heat-sensitive transient receptor potential (TRP) channels. In particular, emodin could significantly increase the mRNA expression of TRPM8 and downregulate TRPV1 mRNA under hot conditions with 39°C in cultured dorsal root ganglion (DRG) neurons (Sui et al., 2010a; Sui et al., 2010b). Moreover, a study to explore the possible mechanism of Dahuang’s antipyretic effect on yeast-induced pyrexia rats found that Dahuang reduced rectal temperature; importantly, it inhibited the fever-induced expression of TRPV1 and enhanced TRPM8 in hypothalamus and DRG. In addition, endogenous pyrogen (PGE2 and cAMP) and antipyretics (AVP) were restored to a balanced state via the treatment of Dahuang (Kong et al., 2014). These findings provide a new perspective for the role of Dahuang with cold property in pyrexia-related disease.

#### Huangqin

Huangqin (the dried root of *Scutellaria baicalensis* Georgi, Lamiaceae), one of the most widely used TCMs, has been prescribed to treat a variety of diseases in many oriental countries, including North Korea, Mongolia, Japan, and Russia (Zhao et al., 2019). One of the characteristics of Huanqin is that it is cold in property according to the theory of TCMs and has the functions of removing the damp-heat, clearing heat, and removing toxicity, stopping bleeding, and tranquilizing fetus (Committee of National Pharmacopoeia, 2015).

According to the cold property and functions of Huangqin, it is a very appropriate choice therapy for patients with hot syndromes such as damp-heat syndrome, heat-toxicity syndrome, and bleeding syndrome. These hot syndromes specifically include symptoms such as gonorrhea, diarrhea, jaundice, cough caused by lung heat, sore throat, hematemesis, hematochezia, and hematuria (Committee of National Pharmacopoeia, 2015). For the treatment of these hot syndromes, Huangqin plays an indispensable role in a number of formulations, which is recorded in many world-renowned monographs of TCM. Huangqin, for instance, exists potent effects on body heat, diarrhea, and dry mouth caused by damp-heat, which comes from *Gegen Qinlian Decoction* in *Shanghan Lun*. Furthermore, Huanglian can also be used to treat a variety of organ dampness-heat syndromes and is especially good at treating cough caused by lung heat. For example, *Qingjin Pill* in *Danxi Xinfa* is effective for cough and sore throat with lung heat only by using Huangqin. The cold property of Huangqin can help stop bleeding, so it has a prominent effect on bleeding syndrome caused by hot. *Huangqin San* composed only of Huangqin can achieve the effect of treating hematemesis and bleeding, which is recorded in *Taiping Shenghui Fang*. Nowadays, Huangqin has been officially registered in Chinese Pharmacopoeia (2015), British Pharmacopoeia (BP 2018), and European Pharmacopoeia (EP 9.0). It is the crucial TCM of numerous renowned traditional Chinese patent medicines and simple preparations, such as *Gegen Qinlian Pills* (used to treat diarrhea, dysentery, influenza, and fever), *Qingqi Huatan Pills* (used to treat cough and sore throat caused by lung heat), *Yinzhihuang Granules* (used to treat jaundice and hepatitis), and *Yiqing Capsule* (used to treat constipation, pharyngitis, tonsillitis, and gingivitis) (Wang Z. L. et al., 2018).

To date, more than 130 compounds have been isolated from Huangqin, most of which are small molecule compounds and a small number of them are polysaccharides. The small molecules can be divided into four types of structures, namely, free flavonoids, flavonoid glycosides, phenylethanoid glycosides, and other small molecules. Among them, the most representative compounds are flavonoids, including baicalin, baicalein, wogonoside, and wogonin (Wang Z. L. et al., 2018). Moreover, the compounds and extracts isolated from Huangqin possess a wide range of pharmacological activities, including antipyretic, antibacterial, antiviral, antioxidative, anticancer, anticonvulsant, anti-inflammatory, antidiabetic, antidepressant, and hepatoprotective effects (Lin et al., 1980; Konoshima et al., 1992; Park et al., 2007; Wang Z. L. et al., 2018; Kong et al., 2019; Fang et al., 2020; Limanaqi et al., 2020). And it is suitable for the treatment of hepatitis, diarrhea, vomiting, hypertension, hyperglycemia, type 2 diabetes, hyperlipidemia, obesity, and nonalcoholic fatty liver (Committee of National Pharmacopoeia, 2015; Fang et al., 2020).

Targeting the pharmacological effects of Huangqin, of note is that the antipyretic effect of it can partially explain its cold property. Just as early as 1980, Lin et al. investigated the effect of Huangqin on thermoregulation in rats and found that intragastric administration of its extract showed a dose-dependent decrease in rectal temperature. And according to the increase in skin temperature, it is estimated that the decrease in body temperature after the application of Huangqin may be caused by skin vasodilation (Lin et al., 1980). Then, Tsai et al. investigated the antipyretic effects of baicalin on rabbits with fever caused by lipopolysaccharide, and the results showed that baicalin may exert its antipyresis by suppressing circulating TNF*a* accumulation and *N*-methyl-d-aspartate (NMDA) receptor-dependent hydroxyl radical pathways in the hypothalamus (Tsai et al., 2006). Furthermore, PGE2 and cAMP, as important neural mediators of fever, also participate in the antipyretic mechanism of baicalin. It was found that baicalin can reverse the change of fever on the contents of PGE2 and cAMP in hypothalamus, and correlation analysis indicated that the contents of PGE2 and cAMP had positive correlations with body temperature in rats (Zhao et al., 2002).

### Section Conclusion

**FIGURE 3:**
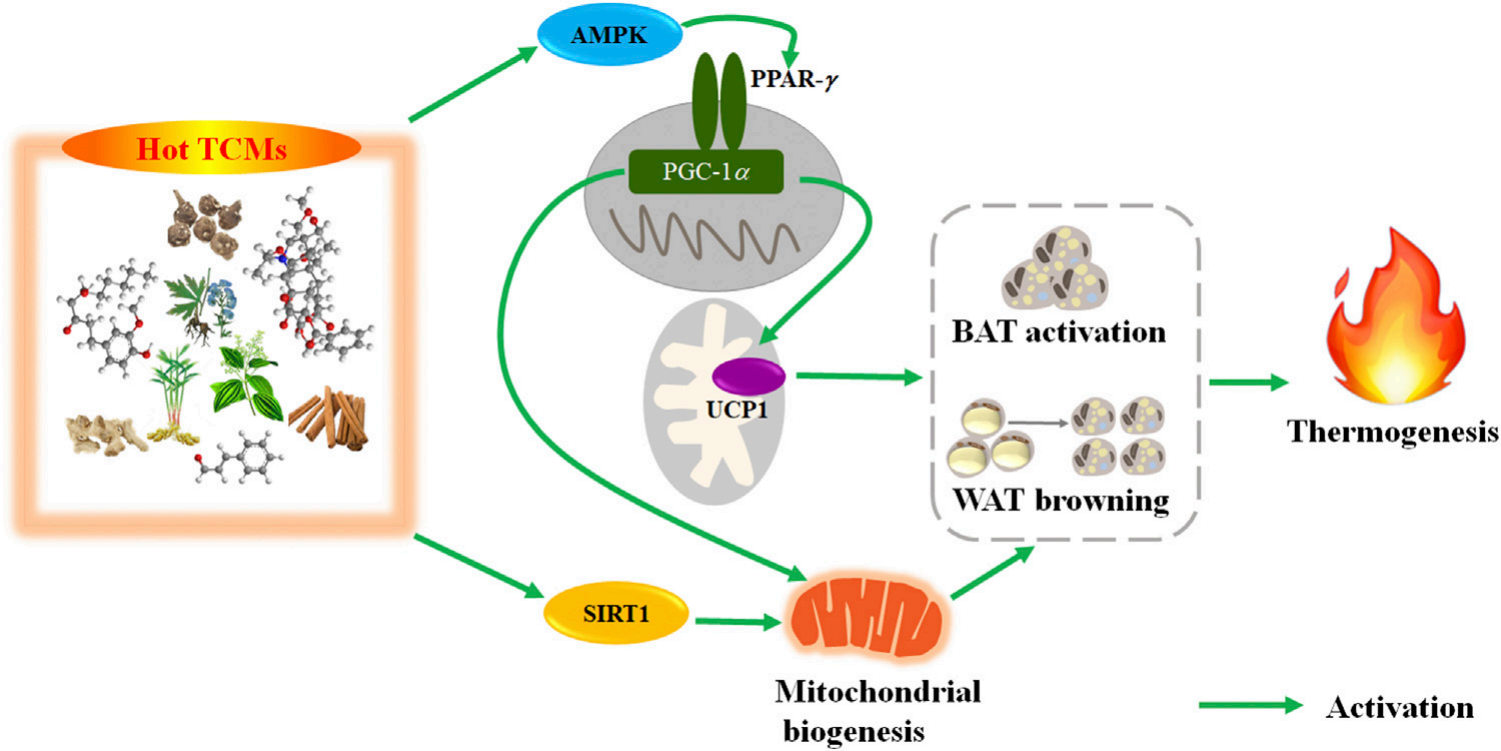
The paradigm summarizes the thermogenesis mechanism of hot TCMs via activating BAT and browning WAT. TCMs, traditional Chinese medicines; PPAR-*γ*, peroxisome proliferator-activated receptor-gamma; PGC-1*α*, peroxisome proliferator-activated receptor-gamma coactivator 1-alpha; UCP1, uncoupling protein 1; SIRT1, silent mating type information regulation 2 homolog 1; BAT, brown adipose tissue; WAT, white adipose tissue.

**FIGURE 4:**
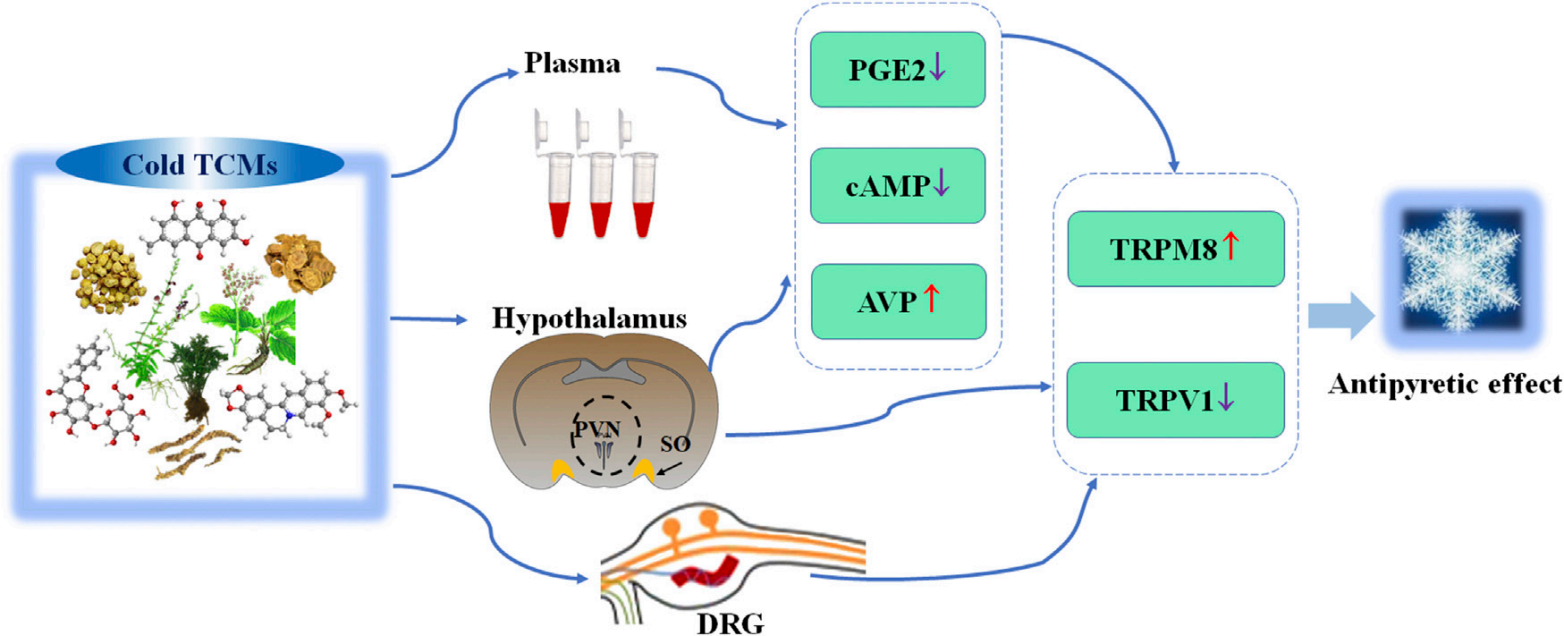
The paradigm summarizes the antipyretic mechanism of cold TCMs via activating TRPV1 and inhibiting TRPM8. TCMs, traditional Chinese medicines; PVN, paraventricular nuclei; SO, supraoptic nucleus; DRG, dorsal root ganglion; PGE2, prostaglandin E2; Camp, cyclic adenosine 3′,5′-monophosphate; AVP, arginine vasopressin; TRPM8, transient receptor potential melastatin 8; TRPV1, transient receptor potential vanilloid 1; ↑, increase; ↓, decrease.

In this section, we introduced the important TCMs with hot property or cold property and their scientific applications. Firstly, we introduced their traditional usage recorded in ancient books and summarized them in Table 1. These traditional usages of typical hot and cold TCMs show the application principle of the theory of cold and hot properties; that is, the TCMs with hot property can be used to treat patients with cold syndrome and the TCMs with cold property can be used to treat patients with hot syndrome. And we found that enhancing *Yang* is the common traditional function of hot TCMs, and clearing heat is the common traditional function of cold TCMs. Then, based on modern research, we summarized their phytochemistry, pharmacological activities, and clinical applications (Table 2). We found that although the compounds of each cold TCM are different, all these representative cold TCMs show antipyretic effect. Similarly, although the components of each TCM are different, all these typical hot TCMs have a promoting effect on thermogenesis. In addition, the mechanisms related to the cold and hot properties of these important TCMs were summarized. Specifically, the mechanism of thermogenesis-promoting effect of these hot TCMs mainly included 1) activating of BAT and browning WAT; 2) promoting energy metabolisms. The BAT activation and WAT browning were the common mechanism of thermogenesis-promoting effect of these hot TCMs. As shown in Figure 3, these hot TCMs activated BAT and induced browning of WAT through the PPAR*γ* (SIRT1-PGC1*α*) pathway, thereby promoting thermogenesis. Similarly, the antipyretic mechanism of these cold TCMs mainly included 1) activating TRPM8 and inhibiting TRPV1; 2) inhibiting proinflammatory cytokines. The common mechanism of antipyretic of these cold TCMs is associated with activation of TRPM8 and inhibition of TRPV1 (Figure 4).

**TABLE 1:** The traditional usage of major TCMs with hot or cold property.

TCMs name	Property	Traditional usage	References
Fuzi	Hot	Revive *Yang* for resuscitation, for prostration, cold sweat, and cold limbs, as well as weak and impalpable pulse Invigorate qi and restore *Yang*, for energy loss, cold extremities, sweating, plus feeble, and impalpable pulse Strengthen spleen *Yang*, for deficient cold in spleen and stomach, cold pain in the abdomen, vomiting, diarrhea, and cold in limbs Restore kidney *Yang*, for dysuria and swelling of the body Dispel coldness and unlock channels, for wind-cold-dampness arthralgia	*Bencao Jingdu* *Shanghan Lun* *Shengji Zonglu* *Taiping Huimin Heji Jufang*
Ganjiang	Hot	Restore *Yang*, for *Yang* depletion syndrome such as prostration, deadly cold hand and foot, and weak pulse caused by the *Yang* deficiency Invigorate spleen and disperse cold, for deficiency of spleen and stomach, cold pain of epigastric abdomen, loss of appetite, reduced diet, vomiting, and diarrhea Warming interior, for dispersing cold and relieve pain Warm lung and resolve fluid retention, for cough and asthma caused by cold, cold in the back, and phlegm retention	*Shanghan Lun* *Jingui Yaolüe* *Heji Ju Fang*
Rougui	Hot	Enhance spleen and kidney *Yang*, for chilly limbs, cold pain in waist and knee, frequent urination, impotence, premature ejaculation, decreased appetite, tiredness, and loose stools Strengthen heart *Yang*, for palpitations, shortness of breath, and chest tightness Dispel cold and relieve pain, for chest pain, heartache, and rheumatism Warm channels to promote blood circulation, for abdominal lumps in women	*Jingyue Quanshu* *Sanyin Fang* *Shoushi Baoyuan* *Beiji Qianjin Yaofang* *Jiyin Gangmu*
Huanglian	Cold	Clear heart fire, for fever, irritability, delirium, dysphoria, palpitation, hematemesis, and bleeding Clear stomach fire, for toothache, redness and swelling of gums, bleeding of teeth, polydipsia, polyuria, polyphagia, emaciation, and fatigue Remove damp-heat, clear heat, and remove toxicity, for dysentery, sores, carbuncles, and furuncles	*Waitai Miyao* *Shanghan Lun* *Jingui Yaolüe* *Taiping Shenghui Fang* *Puji Fang* *Bencao Gangmu* *Waike Zhengzong*
Dahuang	Cold	Defecate stools and remove food, for constipation because of heat accumulation Clear heat, for jaundice caused by damp-heat Cool blood for stopping bleeding, for hematemesis, bleeding, and hemoptysis Clear heat and remove toxicity, for abscess with a pattern of suppuration due to heat exuberance	*Shanghan Lun* *Jingui Yaolüe* *Wenyi Lun*
Huangqin	Cold	Remove damp-heat, for body heat, diarrhea, dry mouth, dysentery, influenza, jaundice, and hepatitis Clear lung heat, for cough and sore throat caused by lung heat Clear away hot, cool blood, and stop bleeding, for bleeding syndrome caused by hot such as hematemesis and hemorrhage	*Shanghan Lun* *Danxi Xinfa* *Taiping Shenghui Fang*

TCMs, traditional Chinese medicines.

**TABLE 2:** The main compounds, pharmacological effects, and clinical applications of major TCMs with hot or cold property.

TCMs name	Property	Major compounds	Pharmacological effects	Clinical applications	References
Fuzi	Hot	Aconitine, mesaconitine, hypaconitine, benzoylmesaconine, benzoylaconitine, benzoylhypacoitine, flavonoids, glucide, saponins, glycosides, fatty acids, ceramides, and uracil	Thermogenesis, the effects on the cardiovascular system, energy metabolism, kidney protection, immune system, anti-inflammation and analgesic action, and antitumor, hypoglycemic, and hypotensive effects	Shock, hypotension, coronary heart disease, chronic heart failure, diarrhea, syncope, neuralgia, joint pain, and rheumatoid arthritis	Yu et al. (2015), Chen et al. (2008), Tang et al. (2012), Sun et al. (2019), Zhao et al. (2020), Konno et al. (1985), Lyu et al. (2008), Sang et al. (2005), He et al. (2018), Deng et al. (2019), Zhao et al. (2012), Zheng et al. (2014), Tan et al. (2014), Yang et al. (2020), Zhang et al. (2017), Huang et al. (2010), Zhou et al. (2015), Zuo et al. (2015)
Ganjiang	Hot	Volatile oils, anthocyanins, tannins, and spicy compounds, namely gingerol, sesquiterpenes and shogaols, alkaloids, and diarylheptanoids	Thermogenesis, hypoglycemic activity, antiatherosclerosis, anti-inflammatory, analgesic, anticancer, antioxidant, and antiaging functions, and the effect on the cardiovascular system and energy metabolism, sedative effect, and antiemetic activity	Dizziness, vomiting caused by cold, vomiting during pregnancy, cough, and asthma	Chrubasik et al. (2005), Semwal et al. (2015), Sharma et al. (2016), Ajish et al. (2015), Li et al. (2016a), Shao et al. (2017), Miyamoto et al. (2015), Samad et al. (2017), Ali et al., 2018, Wang S. et al. (2018), Ho and Chang (2018), Hitomi et al. (2017), Kapoor et al. (2016), Han H. S. et al. (2018), Lee et al. (2019), Vishwakarma et al. (2002), Yu et al. (2012), Wen et al. (2019), Wang W. X. (2016)
Rougui	Hot	Volatile oils such as cinnamaldehyde, nonvolatile compounds including polysaccharides, polyphenols, flavonoids, and other ingredients such as coumarin	Thermogenesis, antioxidant, anti-inflammatory, antibacterial, antidiabetic, antiobesity, antishock, antithrombotic, antigastric ulcer effect, and cardiovascular protection	Cardiovascular diseases, gastrointestinal diseases, diabetes, kidney diseases, rheumatic diseases, and gynecological diseases	Zhang et al. (2019), Liu X. et al. (2018), Anderson et al. (2004), Pardede et al. (2017), Kim (2017), Tamura et al. (2012), Lv et al. (2017), Wang Y. et al. (2018), Zhu et al. (2017), Kwon et al. (2015), Kontogiorgis et al. (2015), Jung et al. (2011), Gao et al. (2015), Hwa et al. (2012), Sebaia et al. (2019), Shinjyo et al. (2020), Zhao et al. (2013b), Chen et al. (2019), Sun et al. (2016)
Huanglian	Cold	Alkaloids such as berberine, palmatine, coptisine, epiberberine, and magnoflorine, as well as organic acids, coumarin, phenylpropanoids, and saccharides	Antipyretic, antibacterial, antiviral, antidiabetic, anticancer, anti-inflammatory, and cardiovascular protective effects	Bacterial-caused diarrhea, hyperlipidemia, diabetes, heart failure, arrhythmia, thrombosis, hypertension, atherosclerosis, and acute coronary syndrome	Perrins (1862), Meng et al. (2018), Tan et al. (2016), Li et al. (2018), Ishikawa et al. (2018), Qin et al. (2020), Kwon and Chan (2020), Hung et al. (2018), Liu et al. (2011), Kumar et al. (2015), Jin et al. (2020), Kong et al. (2004), Dong et al. (2012), Yao et al. (2015), Cicero and Baggioni (2016), Lan et al. (2015), Yao et al. (2015), Li et al. (2016b), Wang K. et al. (2017)
Dahuang	Cold	Anthraquinones such as emodin, emodin methyl ether, aloe emodin, rhein, chrysophanol, etc. and phenolic compounds including catechins, glucose gallates, naphthalenes, sennosides, and stilbenes	Antipyretic, antibacterial, antifungal, antiviral, anti-inflammatory, antioxidant, anticancer, hypoglycemic, immune-enhancing, as well as protecting liver, kidney, and central nervous	Constipation, inflammation, tinnitus, bleeding, pain, dysmenorrhea, tumors, diabetes, gastric ulcers, Parkinson’s disease, Alzheimer’s disease, depression, and severe acute respiratory syndrome	Zargar et al. (2011), Pandith et al. (2014), Pandith et al. (2018), Kong et al. (2014), Esposito et al. (2016), Li X. et al. (2019), Rokaya et al. (2012), Wang et al. (2007), Lakshmi (2016)
Huangqin	Cold	Flavonoids, including baicalin, baicalein, wogonoside, and wogonin, as well as flavonoid glycosides, phenylethanoid glycosides, and polysaccharides	Antipyretic, antibacterial, antiviral, antioxidant, anticancer, anticonvulsant, anti-inflammatory, antidiabetic, antidepressant, and liver protection	Hepatitis, diarrhea, vomiting, hypertension, hyperglycemia, type 2 diabetes, hyperlipidemia, obesity, and nonalcoholic fatty liver	Wang Z. L. et al. (2018), Lin et al. (1980), Konoshima et al. (1992), Park et al. (2007), Kong et al. (2019), Limanaqi et al. (2020), Fang et al. (2020), Committee of National Pharmacopoeia (2015)

TCMs, traditional Chinese medicines.

Major compounds refer to the compounds that have a relatively large percentage in TCMs and have been extensively studied in pharmacology.

After summarizing the traditional function and modern biological mechanism of TCMs, we found the following relationships: the traditional function of enhancing *Yang* of hot TCMs corresponds to thermogenesis-promoting effect. For example, Fuzi (hot in property) can promote thermogenesis by increasing the UCP1 level in BAT, thereby improving hypothermia caused by cold exposure. Similarly, the traditional function of clearing heat of cold TCMs corresponds to antipyretic effect. For example, Dahuang (cold in property) exerts antipyretic effect by activating TRPM8 and inhibiting TRPV1, so as to improve the hot syndrome induced by yeast in rats. These modern biological mechanism studies provide scientific basis for the traditional function of cold and hot TCMs.

## Perspectives

Even though different TCMs may share the same cold property or hot property, the specific mechanisms behind their cold or hot properties are still different. For example, both Fuzi and Rougui are hot TCMs; however, they cannot be used interchangeably in the clinic in most cases. A previous study based on network pharmacology to explore the mechanism of Fuzi and Rougui in the treatment of cardiocerebral vascular diseases showed that, among the 194 targets of Fuzi and Rougui, 45 targets are shared; that is, three-quarters of the targets are different (Li C. et al., 2020). In addition, from a metabolic perspective, Fuzi can promote energy metabolism by increasing the activity of Na^+^-K^+^-ATPase and Ca^2+^-Mg^2+^-ATPase enzymes in hepatocytes, which is closely related to thermogenesis (Yu et al., 2012). However, Rougui shows a significant inhibitory effect on *α*-glucosidase, thereby destroying carbohydrate metabolism and ultimately exerting antidiabetic effects (Shihabudeen et al., 2011). Those studies indicated that the specific mechanisms of the hot property of Fuzi and Rougui are different. Even though progress has been made in revealing this difference, more and more studies are still needed to further identify the specific differences in mechanisms.

In recent years, TCM network pharmacology, first proposed and established by Li et al. (2007), has made a great contribution to the study of hot and cold properties of TCMs including the identification of the cold and hot properties of TCMs and the study of cold and hot syndromes or formulas (Li et al., 2007; Li, 2009; Li et al., 2010; Li and Zhang, 2013; Zhang et al., 2013; Li et al., 2014; Zhang Y. Q. et al., 2016; Zheng et al., 2018). As we all know, in network pharmacology research, animal and human experiments are needed to verify the targets and connections. In animal experiments, metabolomics and other omic tools can be used as powerful means to verify the related targets and connections. Omic tools such as metabolomics have become important techniques for studying the responses of complex organisms to drug or other stimuli. The omic tools can provide complementary information for exploring overall biological function by identifying potential targets, which is consistent with the holistic view of TCM (Wang M. et al., 2017). Therefore, the integration of network pharmacology and omic tools such as metabolomics may be a good strategy to study the mechanism of the effects of cold and hot TCMs.

In addition, gut microbiota has emerged as a new Frontier to understand TCMs (Liu J. et al., 2018; Feng et al., 2018; Feng et al., 2019; Feng et al., 2020). Recent studies have shown that cold or hot exposure can result in marked changes in the composition of gut microbiota and a recent paper also hypothesized that the reshaping of the gut microbiota might serve as the main driver for potentiating energy conservation (Chevalier et al., 2015; Worthmann et al., 2017; Zhang et al., 2018; Li B. et al., 2019; Bo et al., 2019). *Akkermansia muciniphila* is a bacterium that has received a lot of attention because of its health promotion effect. Studies have demonstrated that *Akkermansia muciniphila*, as an energy sensor, is positively related to the markers of the beginning, like uncoupling protein-1 (UCP-1) (Derrien et al., 2008; Everard et al., 2013; Schneeberger et al., 2015; Liu et al., 2017). Further evidence indicates that cold exposure changes the diversity and composition of the gut microbiota in mice, resulting in alters in the production of microbial metabolites (Ziętak et al., 2016; Worthmann et al., 2017; Li B. et al., 2019; Wang D. et al., 2020). Among these metabolites, the production of short-chain fatty acids (SCFAs) is found to be significantly increased in cold environments. Moreover, isotope tracing experiments have shown that SCFAs are distributed in multiple tissues (including the brain, WAT, and BAT) and metabolized in the tricarboxylate (TCA) cycle (Zhang et al., 2018; Li B. et al., 2019). Notably, a recent study showed that butyrate (SCFA) increases the thermogenesis in BAT and WAT by activating lysine-specific demethylase 1 (LSD1) (Wang D. et al., 2020). In addition, bile acids (BAs), the metabolites of gut microbiota, play an important role in thermogenesis (Teodoro et al., 2014; Zietak and Kozak, 2016). Recently, research showed that bile acid can shape the gut microbiome and promote adaptive thermogenesis in a cold environment (Worthmann et al., 2017). Taken together, those studies have shown that gut microbiota and gut microbiota metabolites play a key role in regulating thermogenesis during cold or hot exposure. Thus, it can be speculated that the cold and hot properties of TCMs might have an important relationship with gut microbiota metabolites. Correspondingly, the integration of 16S rRNA gene sequencing and metabolomics, a multiomics combination mode, can be considered in the future to study the cold and hot properties of TCMs.

## Conclusion

Cold and hot properties of TCMs have played and will still play important roles in guiding TCM practitioners to combating human diseases. From ancient times to the present, people have been exploring the mystery of cold and hot properties of TCMs. In ancient times, the identification of cold and hot properties mainly depends on the healthy human’s direct feeling on a given TCM or the effects of TCMs on patients with cold or hot syndrome. Nowadays, some animal models (such as collagen-induced arthritis rats) and advanced technologies (such as network pharmacology) have been used to identify the cold and hot properties of TCMs. In recent years, phytochemical and pharmacologic research of cold and hot TCMs have indicated that although the chemical components are different, the representative cold TCMs all have antipyretic effects, and the typical hot TCMs all can promote thermogenesis. In addition, the studies on the mechanism related to the cold and hot properties have demonstrated that activating TRPM8 and inhibiting TRPV1 are the common mechanisms of the antipyretic effect of cold TCMs, and BAT activation and WAT browning are the common mechanisms of hot TCMs to promote thermogenesis. Although some progress has been made in understanding the cold and hot properties of TCMs, more and more studies are still needed to further explore the mechanism differences of TCMs with the same property.

## Author Contributions

WF and CP conceived of and proposed the idea. JL, WF, and CP reviewed literature. JL, WF, and CP contributed to writing, revising, and proofreading the review. All authors read and approved the final manuscript.

## Funding

This work was supported by the National Natural Science Foundation of China (nos. 81891010, 81891012, 81630101, and U19A2010) and Science and Technology Ministry of China (2108ZX09721001-008).

## Conflict of Interest

The authors declare that the research was conducted in the absence of any commercial or financial relationships that could be construed as a potential conflict of interest.
